# When Do Medical Doctors Recommend Less Expensive Medication Prescriptions in U.S. Adults 50 Years or Older?

**DOI:** 10.1093/geroni/igaa057.268

**Published:** 2020-12-16

**Authors:** Jose Aravena

**Affiliations:** School of Public Health, Yale University, New Haven, Connecticut, United States

## Abstract

The US is the country with the greatest average spending for prescription drugs per person (US$ 1,200). 85.0% of older people consume prescription medications, with high rates of polypharmacy. The aim was to analyze the factors related to the recommendation of a less expensive prescription by a medical doctor in US older adults. A cross-sectional analysis using the data from The National Poll on Healthy Aging (NPHA) 2017 was conducted, with a total sample of 1666 adults age 50 to 80 residing in the US. People were asked if they have received a less expensive prescription by a medical doctor in the last two years (yes/no). Sociodemographic and health variables, active patient medication-cost behaviors, and doctor active medication-costs actions were measured as covariates. Weighted and stratified by region logistic regression model was conducted in a 70% random sample. The model was validated in the 30% remaining using ROC curve and AUC. In the parsimonious model, ≥4 visits to the doctor (OR=2.06, 1.33 - 3.18), perception of medication costs as a burden (OR=1.76, 1.25 - 2.47), the doctor talked about medication costs (OR=5.54, 3.90 - 7.88), doctor awareness of medication costs (OR=1.81, 1.34 - 2.46), and being Non-Hispanic Black (OR=1.90, 1.20 - 3.03) were linked to a higher odd to receive a less expensive prescription. The model presented a moderate-high fit (AUC:0.71; sensitivity:84.4%, specificity:49.8%). Awareness and training in the active prescription of less expensive medications by the medical doctor seem fundamental to reduce drug costs burden in older adults.

